# Phylogenetic Analyses of Xanthomonads Causing Bacterial Leaf Spot of Tomato and Pepper: *Xanthomonas euvesicatoria* Revealed Homologous Populations Despite Distant Geographical Distribution

**DOI:** 10.3390/microorganisms7100462

**Published:** 2019-10-16

**Authors:** Upasana Dhakal, Shefali Dobhal, Anne M. Alvarez, Mohammad Arif

**Affiliations:** Department of Plant and Environmental Protection Sciences, University of Hawaii at Manoa, Manoa, HI 96822, USA; upasana@hawaii.edu (U.D.); shefali@hawaii.edu (S.D.); alvarez@hawaii.edu (A.M.A.)

**Keywords:** *Xanthomonas euvesicatoria*, multilocus sequence analysis (MLSA), ELISA, *Xanthomonas* genomes, phylogenetics, population genetics, phytobacteria, bacterial leaf spot

## Abstract

Bacterial leaf spot of tomato and pepper (BLS), an economically important bacterial disease caused by four species of *Xanthomonas* (*X. euvesicatoria* (*Xe*), *X. vesicatoria* (*Xv*), *X. gardneri* (*Xg*), and *X. perforans* (*Xp*)), is a global problem and can cause over 50% crop loss under unfavorable conditions. Among the four species, *Xe* and *Xv* are prevalent worldwide. Characterization of the pathogens is crucial for disease management and regulatory purposes. In this study, we performed a multilocus sequence analysis (MLSA) with six genes (*hrcN*, *dnaA gyrB*, *gapA*, *pdg*, and *hmbs*) on BLS strains. Other *Xanthomonas* species were included to determine phylogenetic relationships within and among the tested strains. Four BLS species comprising 76 strains from different serological groups and diverse geographical locations were resolved into three major clades. BLS xanthomonads formed distinct clusters in the phylogenetic analyses. Three other xanthomonads, including *X. albilineans*, *X. sacchari*, and *X. translucens* pv. *undolusa* revealed less than 85%, 88%, and 89% average nucleotide identity (ANI), respectively, with the other species of *Xanthomonas* included in this study. Both antibody and MLSA data showed that *Xv* was clearly separated from *Xe* and that the latter strains were remarkably clonal, even though they originated from distant geographical locations. The *Xe* strains formed two separate phylogenetic groups; *Xe* group A1 consisted only of tomato strains, whereas *Xe* group A2 included strains from pepper and tomato. In contrast, the *Xv* group showed greater heterogeneity. Some *Xv* strains from South America were closely related to strains from California, while others grouped closer to a strain from Indiana and more distantly to a strain from Hawaii. Using this information molecular tests can now be devised to track distribution of clonal populations that may be introduced into new geographic areas through seeds and other infected plant materials.

## 1. Introduction

Bacteria cause many important diseases in cultivated and wild plants throughout the world [1]. The genus *Xanthomonas* consists of many pathogens of economic importance that cause diseases in plants of over 200 families [1,2]. Bacterial leaf spot of tomato and pepper (BLS) is caused by four species of *Xanthomonas*, (*X. euvesicatoria*, *X. vesicatoria*, *X. perforans*, and *X. gardneri*) and is a worldwide problem in tomato and pepper production [3]. Approximately 17.7 million tons of tomato and 34.5 million tons of pepper were produced in 2016 (FAO 2016), and seed production is an essential component of these industries. Bacterial leaf spot is a seed-borne disease and can be destructive in warm and humid conditions, causing up to 50% of the yield loss in favorable conditions [4]. Xanthomonads may enter through natural openings, such as stomata, causing localized leaf spots, or through hydathodes resulting in vascular infection.

Formerly, all xanthomonads causing BLS were recognized as *X. campestris* pv. *vesicatoria*. In 1994, Stall et al. described two distinct groups within *X. campestris* pv. *vesicatoria* based on carbon substrate utilization, fatty acid profiles, starch hydrolysis, and ability to degrade pectin. The two groups had less than 50% DNA homology with DNA–DNA hybridization [5]. Fatty acid profiles, protein profiles, carbon substrate utilization, and ELISA using a panel of monoclonal antibodies were used to characterize diversity within a worldwide collection of BLS strains [5,6]. ELISA differentiated former *X. campestris* pv. *vesicatoria* strains from pepper and tomato into six serovars; three serovars were within two groups. Groups A and B were further differentiated by protein profile analysis and amylolytic activity [6]. In 1995, the BLS xanthomonads were further separated into two species, *X. vesicatoria* and *X. axonopodis* pv. *vesicatoria* based on DNA–DNA hybridization studies [7]. Later, Jones et al. [3] reclassified the BLS xanthomonads into four species, namely, *X. vesicatoria*, *X. euvesicatoria*, *X. gardneri*, and *X. perforans*, based on a thorough study of phenotypic characteristics, including antibody patterns, protein profiles, and pulse field gel electrophoresis. *X. gardneri* and *X. perforans* are newly described pathogens of tomato and pepper, while *X. vesicatoria* and *X. euvesicatoria* are of historical importance and are prevalent worldwide. Production of indistinguishable symptoms on a common host makes a visual diagnosis difficult for BLS xanthomonads; thus, researchers rely on molecular tools, such as sequencing, multilocus sequence typing (MLSA), and loop-mediated isothermal amplification to identify the species [8,9]. Characterization of genetic diversity is critical for management and regulatory purposes, and accurate identification of key differences and possible changes in pathogen populations [10] facilitates deployment of resistant cultivars, which is one sustainable approach to disease management [11]. Parkinson et al. [12,13] established *gyrB* gene as a simple and rapid method for phylogenetics and diagnostics of xanthomonads, including BLS-causing *Xanthomonas* species. MLST is commonly used to characterize the genetic diversity of pathogens based on selected loci within the genome [14,15], and this knowledge helps to identify possible new sources of inoculum and deploy disease management options.

An *X. euvesicatoria* strain identical to type strain NCPPB 2968 was the dominant pathogen causing BLS in eastern Australia, whereas *X. vesicatoria* strains, though fewer in number, formed two separate groups in phylogenetic studies [8]. Two groups within the *X. vesicatoria* population were also reported from Central Ethiopia [16]. Timilsina et al. [17] conducted the study on xanthomonads causing BLS by using strains collected from different geographical locations. Three haplotypes of *X. euvesicatoria* were identified and most of the strains were identical to type strain 85-10 [17]. Three haplotypes also were identified within *X. vesicatoria*, which had a smaller representation (nine strains) in the collection [17]. Most recently, Roach et al. [8,18] studied the diversity of recently collected xanthomonads from Australia causing BLS based on MLST and genome analyses.

Movement of pathogens in infected planting materials and seeds contributes to the spread of bacterial pathogens throughout the world and is significant in the case of BLS of tomato and pepper, which are seed-borne diseases. Nevertheless, both geographic isolation and selection pressure in specific locations shape the evolution and diversity within geographically isolated groups of pathogens. Most of the studies dealing with the diversity of *X. euvesicatoria* and *X. vesicatoria* are based on a few common genes [16,17]. The increasing availability of whole genome sequences of *X. euvesicatoria* and *X. vesicatoria* in public databases and new genome comparison tools allow genome-wide comparison and selection of robust markers (genes) for population genetic studies [14]. More studies with diverse strains from worldwide origins and new markers with higher potential to detect discrepancies within species are needed for a detailed analysis of the genetic diversity of *X. euvesicatoria* and *X. vesicatoria*. The rapid advancement in molecular technologies, including accessibility, affordability of sequencing facilities, and next-generation sequencing has now enabled detailed studies of diversity within and among plant pathogens [10,14,18]. Additional genotypes within each species of BLS xanthomonads have been discovered and multiple populations have been found at the same location [16,17].

The objective of this study was to characterize the diversity between and within *X. euvesicatoria* and *X. vesicatoria* strains collected in different years (mostly from 1960s to 1990s) from diverse worldwide geographical locations. A comparative genomic analysis was undertaken to select appropriate genes for better resolution of the population structure.

## 2. Materials and Methods

### 2.1. Bacterial Strains and DNA Extraction

The seventy-six strains used in this study were collected from different geographical locations including Australia, Taiwan, South America, California, Florida, Indiana, and Hawaii at different time intervals; details of the bacterial strains are listed in Table 1. The strains were isolated from infected pepper and tomato. Cultures stored at −80°C (TB medium) were grown on tetrazolium chloride medium (5 g peptone, 2.5 g dextrose, 8.5 g agar, 0.5 mL 1% TZC in 500 mL of distilled water) and purified by subculturing from the single colony. DNA was extracted from purified bacterial cultures using Wizard Genomic DNA Purification Kit (Promega, Madison, WI, USA) following the manufacturer’s instructions. The isolated DNA was quantified using NanoDrop™ 2000/c spectrophotometer (Thermo Fisher Scientific, Waltham, MA, USA).

### 2.2. Gene Selection and Primer Design

A total of six genes were selected based on their ability to discriminate the populations. The replication initiation factor (*dnaA*), type I glyceraldehyde-3-phosphate dehydrogenase (*gapA*), and DNA topoisomerase (ATP-hydrolyzing) subunit B (*gyrB*) genes are commonly used for population genetics studies of *Xanthomonas* species and were selected for this study after initial analysis [10,11,12,13]. Three genes were selected after genome-wide analyses of four BLS xanthomonads; genomes were retrieved from the National Center for Biotechnology Institute GenBank genome database (NCBI, Bethesda, MD). Whole genomes of *X. euvesicatoria* (NZ_CP018467, NZ_CP017190, and NC_007508), *X. vesicatoria* (NZ_CP018470 and NZ_CP018725) *X. gardneri* (NZ_CP018728 and NZ_CP018731), and *X. perforans* (NZ_CP019725 and NZ_CP018475) were aligned using Mauve [20] and potential genomic regions for population genetics studies were examined. Conserved genes among *X. euvesicatoria*, *X. vesicatoria*, *X. perforans*, and *X. gardneri* with the ability to detect interspecific and intraspecific discrepancies were selected. The *hrcN* gene, conserved in all four BLS xanthomonads but with the ability to discriminate these four species, was selected for identity confirmations and included in the population genetics study [9]. All three complete genomes of *X. euvesicatoria* were downloaded from NCBI GenBank database and compared; however, these genomes showed high homology and were not helpful for selection of genes that discriminate *X. euvesicatoria* populations. So, twelve *X. euvesicatoria* shotgun genome sequences were retrieved from the NCBI GenBank genome database and were aligned using progressive Mauve; the aligned files were exported to Geneious (version 10.2.3) and screened for the regions that could differentiate *X. euvesicatoria* populations. The pyruvate dehydrogenase (acetyl-transferring) (*pdg*) and hydroxymethylbilane synthase (*hmbs*) genes were specifically selected to differentiate *X. euvesicatoria* populations. Primers for *gapA*, *gyrB*, *pdg*, and *hmbs* were designed based on the sequence alignments of individual genes extracted from whole genomes of *X. euvesicatoria*, *X. vesicatoria*, *X. perforans*, and *X. gardneri;* conserved regions were used for primer design, which was done using primer3 software version 0.4.0 [21]. Previously reported primers were used for *dnaA* [19] and *hrcN* gene regions (Table 2) [9]. A total of 8 primer sets were used for the amplification of six target genes. Details of the primers are listed in Table 2. Due to the high nucleotide diversity in the *hmbs* gene, it was difficult to design a single primer that could amplify all BLS xanthomonads to give a desired product size (600–1000 bp). Therefore, two pairs of primers were designed for *hmbs* gene. Primer Hmbs-F and Hmbs-R were designed to target *X. euvesicatoria* and *X. perforans.* Likewise, Hmbs-F2 and Hmbs-R2 were designed to target *X. vesicatoria* and *X. gardneri*. A single primer set was used for amplification of the remaining genes for all four species. Five strains of *X. vesicatoria* gave no product with Hmbs-F2 and Hmbs-R2. Therefore, primers Hmbs-F10 and Hmbs-R10 were designed based on the sequence alignment of *X. vesicatoria* obtained with Hmbs-F2 and Hmbs-R2 primers along with *hmbs* sequences retrieved from the published whole genomes of *X. vesicatoria*.

### 2.3. PCR, Sequencing, and Identity Confirmation

PCR reactions were carried out in 20 μL volumes in a T100 Thermocycler (Bio-Rad, Hercules, CA, USA). One μL of each forward and reverse primer (5 µm), 10 μL of Gotaq Green master mix (Promega), 1 μL genomic DNA, and 7 μL sterile distilled water was used for each reaction. One microliter of sterile water was added to the negative control instead of DNA. Cycling conditions for *hrcN*, *gyrB*, *gapA*, *pdg*, and *hmbs*: initial denaturation for 5 min at 94 °C, followed by 35 cycles at 94 °C for 20 s, 60 °C for 30 s, and 72 °C for 1 min; this was followed by a final extension step at 72 °C for 3 min. For primer Hmbs-F10, all conditions were the same except that annealing was performed at 52 °C for 1 min. For *dnaA* primer set, cycling conditions consisted of initial denaturation at 94 °C for 5 min, followed by 35 cycles at 94 °C for 30 s, 60 °C for 1 min, an extension at 72 °C for 30 s, and a final extension at 72 °C for 10 min. To confirm the amplification of the target region, 5 μL of PCR product was electrophoresed on 1.5% agarose gel for 90 min and visualized under UV light (FOTODYNE Incorporated, Hartland, WI, USA). The PCR product was purified by mixing 5 μL of PCR product with 2 μL ExoSAP-IT™ (Affymetrix Inc, Santa Clara, CA, USA); the mixture was incubated at 37 °C for 15 min followed by 80 °C for 15 min [22]. Sequencing was performed at GENEWIZ facility (Genewiz, La Jolla, CA, USA). Obtained sequences of each strain were aligned using Geneious and manually edited to correct the sequencing errors. Finally, manually edited and corrected sequences were compared with the publicly available NCBI GenBank nucleotide and genome databases using BLASTn tool to confirm the identity of each isolate used in this study.

### 2.4. Phylogenetic Analysis of X. euvesicatoria and X. vesicatoria

Multiple alignments of the individual or concatenated sequences were used to generate the phylogenetic trees using CLC Genomics Workbench 12.0.3 and R studio 3.4.3 [23]. Multiple sequence alignments were obtained using multiple sequence comparisons by the log-expectation (MUSCLE) alignment tool in Geneious. Phylogenetic analysis was conducted using package ape and CLC Genomics Workbench to obtain a neighbor-joining tree. Pairwise distance was calculated among the DNA sequences using “K80” evolutionary model. From the sequence data generated in this study, six individual trees were obtained for the genes *hrcN*, *dnaA*, *gyrB*, *gapA*, *pdg*, and *hmbs*. In addition to this, sequences for *dnaA*, *gyrB*, *gapA*, *pdg*, and *hmbs* were concatenated and a tree was generated. Confidence for the branches was calculated by bootstrapping the tree 1000 times using the function “boot.phylo” in the R package, ape version 5.0 [24], and CLC Genomics Workbench. The obtained tree was plotted and annotated using the package ggtree version 1.10.4 [25] and CLC Genomics Workbench. Bootstrap values were plotted as node labels.

### 2.5. Phylogenetic Analysis of X. euvesicatoria along with Other Xanthomonas Species

To determine the phylogenetic position of *X. euvesicatoria* and *X. vesicatoria* relative to other plant pathogenic xanthomonads and vice versa, a total of 10 genes were retrieved from the whole genomes of plant pathogenic *Xanthomonas* species including sequences for 5 genes used in this study (*dnaA*, *gyrB*, *gapA*, *pdg*, and *hmbs*) and 5 other genes not used in this study (*lacF*, *fusA*, *gltA*, *dnaK*, and *16S*). All the reference whole genomes or shotgun sequences of bacterial strains (Table 3) were retrieved from the NCBI GenBank. Sequences for nine housekeeping genes (*dnaA*, *gyrB*, *gapA*, *pdg*, *hmbs*, *lacF*, *fusA*, *gltA*, *dnaK*) were concatenated and a NJ (Neighbor-joining) tree was generated to draw a better picture of the phylogeny for plant pathogenic xanthomonads. A separate tree was plotted with 16s sequences for all *Xanthomonas* species. Finally, *dnaA*, *gyrB*, *gapA*, *pdg*, and *hmbs* sequences for strains used in this study were aligned along with reference sequences of other *Xanthomonas* species to obtain a tree. Neighbor-joining trees were generated and annotated following the previously mentioned procedure.

### 2.6. ELISA Analysis

Five monoclonal antibodies previously produced in our laboratories were used to assess the diversity in BLS xanthomonads isolated from pepper and tomato. Mabs Xv1, Xv3, Xv5, Xv 7, and Xv15 were produced following the protocol by Alvarez et al. [26]; Mab Xv8 was generated and evaluated by Bouzar et al. [6]. The immunogen, clone number, and subclass for each MAb is listed in Table 4.

All the strains listed in Table 1 except *X. gardneri* and *X. perforans* were tested by ELISA to confirm the initial identification using genus specific MAbs X1 and X11 [26], and later with the panel of MAbs generated to differentiate strains earlier classified as *X. campestris* pv. *vesicatoria*. Positive and negative reactions were recorded as 1 and 0, respectively. Results were used to get the matrix of binary number, strains on vertical axis, and antibodies on the horizontal axis. The matrix was exported to software R 3.4.3, pairwise distance was calculated, and a neighbor-joining tree was constructed following the above-mentioned procedure.

## 3. Results

### 3.1. PCR Amplification, Sequencing, and Identity Confirmation

After comparing the *hrcN* gene sequences against the NCBI GenBank nucleotide and genome databases, the identity of 58 and 14 strains were confirmed as *X. euvesicatoria* and *X. vesicatoria*, respectively. The identities of two strains received as *X. perforans* and *X. gardneri* were also reconfirmed by sequencing and BLASTn. Primers designed to amplify the *hrcN*, *dnaA*, *gyrB*, *gapA*, and *pdg* gene regions successfully amplified all the strains. However, for the *hmbs* gene, five *X. vesicatoria* strains, A3618, A3788, A1696, A1703, and A1887, did not amplify with the primer set Hmbs-F2 and Hmbs-R2. Successful amplification of the *hmbs* gene for four strains (A3618, A3788, A1696, and A1887) was achieved with a new primer set Hmbs-F10 and Hmbs-R10. Despite the effort to use alternate primers, strain A1703 failed to amplify with all possible combinations of primers sets Hmbs-2 and Hmbs-10. This strain was thus removed from the concatenated analysis.

### 3.2. Phylogenetic Analysis

The sequences were manually edited for higher accuracy; poor quality sequences from both 3′ and 5′ ends were removed. After error corrections, 729-, 718-, 702-, 796-, 676-, and 565-bp sequences were obtained for *hrcN*, *dnaA*, *gyrB*, *gapA*, *pdg*, and *hmbs* genes, respectively. All the sequences were submitted to the NCBI GenBank and the accession numbers are listed in Table S1. All six genes used for the population genetics grouped *X. euvesicatoria*, *X. vesicatoria*, *X. gardneri*, and *X. perforans* into distinct clusters (Figures S1–S6). The genes *hrcN*, *gyrB*, and *gapA* were unable to differentiate populations within *X. euvesicatoria* (Figures S1, S3 and S4) and there was no congruence among different groups of *X. vesicatoria* obtained with individual genes (Figures S1, S3 and S4). Phylogenetic analysis with *dnaA* differentiated *X. euvesicatoria* into a major group and a small subgroup within this group; however, *X. vesicatoria* showed high divergence among the strains (Figure S2). Four *X. euvesicatoria* strains that formed a small subgroup, A1718, A3799, A1926, A3621, differed from all other *X. euvesicatoria* strains by single nucleotide for *dnaA* gene. Phylogenetic analysis with *pdg* gene gave four *X. vesicatoria* groups, while *hmbs* gene clustered the *X. vesicatoria* strains into three groups (Figures S5 and S6).

**Figure 1:**
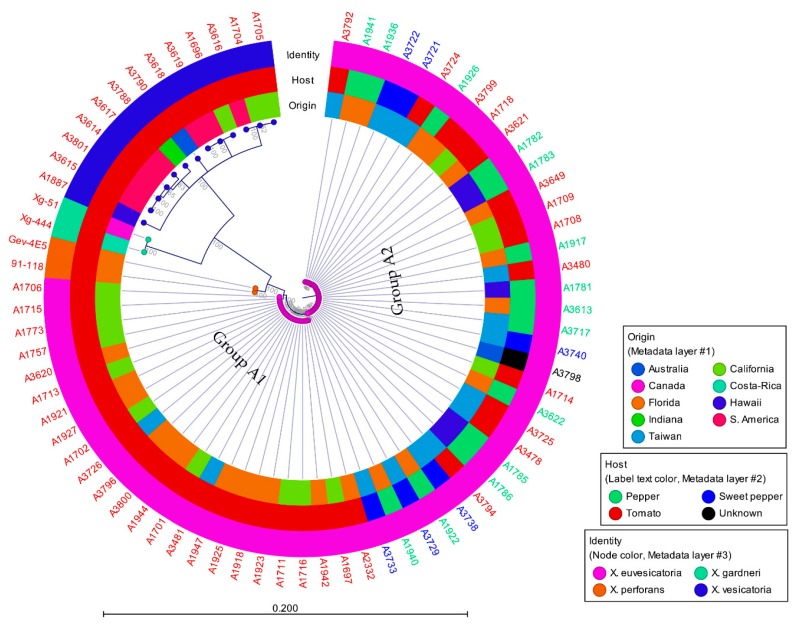
Concatenated phylogenetic analysis of *Xanthomonas euvesicatoria* and *X. vesicatoria* derived using genes EscN/YscN/HrcN family type III secretion system ATPase (*hrcN*), chromosomal replication initiator factor (*dnaA*), DNA topoisomerase (ATP-hydrolyzing) subunit B (*gyrB*), type I glyceraldehyde-3-phosphate dehydrogenase (*gapA*), hydroxymethylbilane synthase (*hmbs*), and pyruvate dehydrogenase (*pdg*). *X. vesicatoria* strain A1703 was removed from the analysis due to no amplification with *hmbs* gene primers. Numbers on the nodes represent bootstrap values and are presented as percentage of 1000 replicates.

All six genes were concatenated and produced a ~4186 bp long fragment. In phylogenetic analysis, *X. vesicatoria* formed a single tight cluster with small subgroups, while *X. euvesicatoria* formed two major groups (Figure 1). Two strains of *X. gardneri* and two strains of *X. perforans* grouped separately from *X. euvesicatoria* and *X. vesicatoria*, where *X. gardneri* was close to *X. vesicatoria* and *X. perforans* was close to *X. euvesicatoria*. *X. euvesicatoria* resolved into two clades, while *X. gardneri*, *X. perforans*, and *X. vesicatoria* comprised the third clade. *X. euvesicatoria* group A1 was well separated from group A2, with strong bootstrap support of 100. *X. euvesicatoria* groups A1 and A2 were different at 16 nucleotide positions. Group A1 and group A2 differed by seven 7 nucleotides for *hmbs* and nine nucleotides for *pdg*, where the differences were spread across the gene. Group A2 and a small subgroup within group A2 were different from each other by a single nucleotide in *dnaA* gene. Overall, genes *hmbs* and *pdg* were more variable. All the *X. euvesicatoria* strains in group A1 were from tomato and most of the strains in group A2 were from pepper, but it also had strains from tomato. *X. euvesicatoria* strains grouped together irrespective of their geographic origin. Color-coded matrix showing pairwise percentage identity of *X. euvesicatoria, X. vesicatoria, X. perforans*, and *X. gardneri* strains shown in Figure S7.

### 3.3. Phylogenetic Position of X. vesicatoria and X. euvesicatoria Relative to Other Xanthomonads

**Figure 2:**
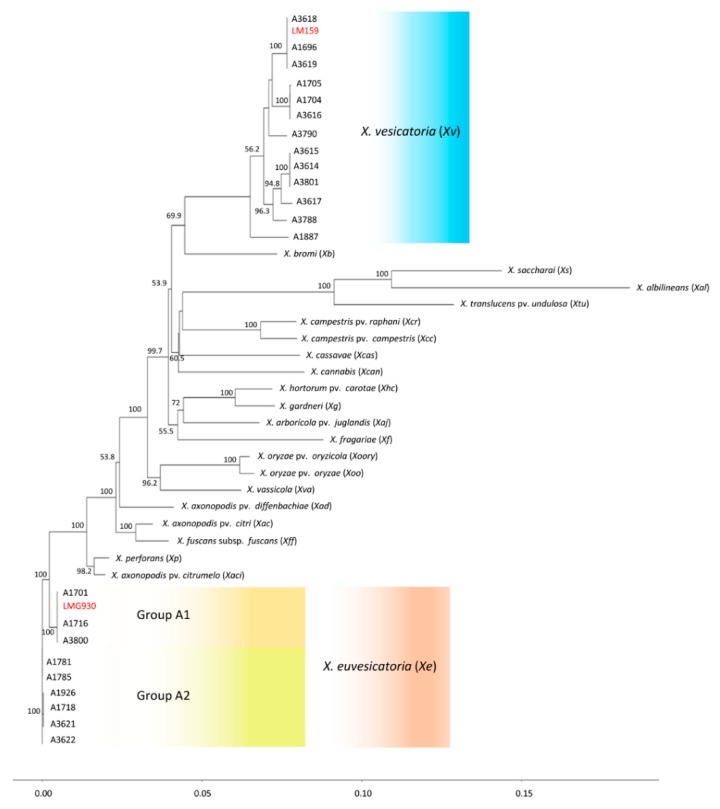
Phylogenetic position of *Xanthomonas euvesicatoria*, *X. vesicatoria*, and nineteen other plant pathogenic xanthomonads relative to each other. The phylogenetic tree was obtained using concatenated gene sequences for chromosomal replication initiator factor (*dnaA*), type I glyceraldehyde-3-phosphate dehydrogenase (*gapA*), DNA topoisomerase (ATP-hydrolyzing) subunit B (*gyrB*), hydroxymethylbilane synthase (*hmbs*), and pyruvate dehydrogenase (*pdg*) genes. Numbers on the nodes represent bootstrap values and are presented as percentage of 1000 replicates. The scale bar at the bottom shows the distance between strains of other species and strains of *X. euvesicatoria* and *X. vesicatoria* sequenced in this study. Sequences for other *Xanthomonas* species were extracted from the genomes listed in Table 3. LM159 and LMG930 are reference strains for *X. vesicatoria* and *X. euvesicatoria*, respectively.

**Figure 3:**
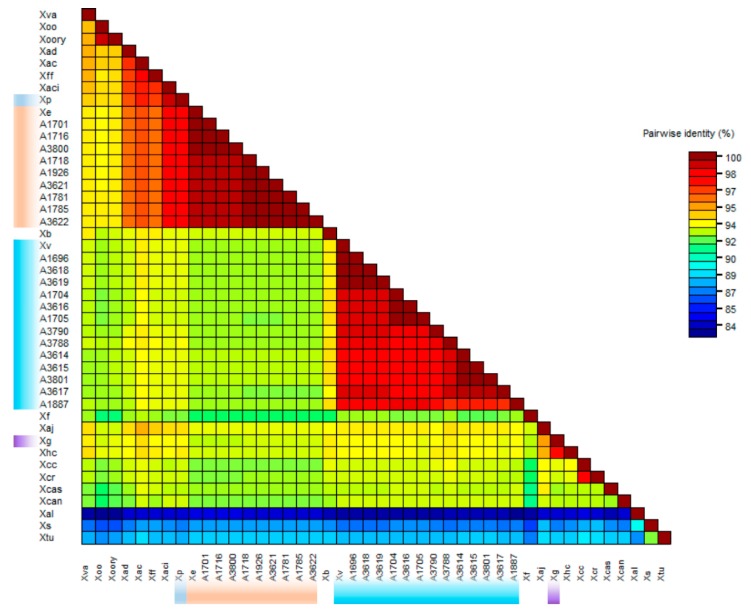
Color-coded matrix showing pairwise percentage identity of *Xanthomonas euvesicatoria*, *X. vesicatoria* with other species of *Xanthomonas* for chromosomal replication initiator factor (*dnaA*), type I glyceraldehyde-3-phosphate dehydrogenase (*gapA*), DNA topoisomerase (ATP-hydrolyzing) subunit B (*gyrB*), hydroxymethylbilane synthase (*hmbs*), and pyruvate dehydrogenase (*pdg*) genes. The information about the strains is provided in Table 1.

Sequences for five genes (*dnaA*, *gyrB*, *gapA*, *hmbs*, and *pdg*) of all *X. vesicatoria* strains and nine *X. euvesicatoria* representative strains from group A1 (A1701, A1716, A3800), A2 (A1781, A1785, A3622), and a subgroup within group A2 (A1926, A1718, A3621), along with the other *Xanthomonas* species (sequences of *dnaA*, *gyrB*, *gapA*, *hmbs*, and *pdg* genes were extracted from the genome or shotgun sequences, NCBI GenBank genome database), were used to determine the relative position of *X. euvesicatoria* and *X. vesicatoria* in a phylogenetic tree (Figure 2 and Figure 3). Gene *gyrB* showed several gaps in sequence alignment. Phylogenetic analysis of the concatenated sequences resolved the plant pathogenic xanthomonads into eight well supported clades (Figure 2). *X. vesicatoria* clustered together in a clade with *X. bromi*; *X. vesicatoria* reference strain LM159 was identical to strains A3618, A1696, and A3619, and formed a subgroup within the *X. vesicatoria* clade. *X. saccharai*, *X. albilineans*, and *X. translucens* pv. *undulosa* formed the second clade. *X. campestris* pv. *raphani*, *X. campestris* pv. *campestris*, *X. cassavae* and *X. cannabis* formed the third clade. The fourth clade was comprised of *X. hortorum* pv. *carotae*, *X. gardneri*, *X. arboricola* pv. *Juglandis*, and *X. fragariae*, whereas *X. oryxae* pv. *oryzae*, *X. oryzae* pv. *oryzicola*, and *X. vassicola* grouped together to form the fifth clade. *X. axonopodis* pv. *citri* and *X. fuscans* subsp. *fuscans* formed sixth clade, while *X. perforans* and *X. axonopodis* pv. *citrumelo* were in the seventh clade. *X. euvesicatoria* formed the eighth clade. The *X. euvesicatoria* reference strain LMG930 was identical to *X. euvesicatoria* group A1 strains and was 98%, 97.9%, 96%, 93%, and 92.6% identical to *X. axonopodis* pv. *citrumelo*, *X. perforans*, *X. campestris* pv. *dieffenbachiae*, *X. gardneri*, and *X. vesicatoria*, respectively. Reference strain LMG 930 shared only 84.7%, 85.5%, and 87.9% nucleotide identity with *X. albilineans*, *X. translucens*, and *X. sacchari*, respectively. Similarly, *X. vesicatoria* reference strain LM159 was most closely related to *X. bromi* (94%) and was least close to *X. albilineans* (83.6%). *X. sacchari* and *X. translucens* pv. *undulosa* shared only 87.2% and 88.3% average nucleotide identity with *X. vesicatoria* reference strain LM159. *X. campestris* pv. *campestris* and *X. campestris* pv. *raphani* were 97.8% identical. The xanthomonads, *X. sacchari*, *X. albilineans*, and *X. translucens* pv. *undulosa* in the second clade were very different than the remaining strains. *X. albilineans* was less than 85% similar, *X. sacchari* shared less than 88% average nucleotide identity, and *X. translucens* pv. *undulosa* was less than 89% identical to the other xanthomonads (species other than X. *sacchari*, *X. translucens* pv *undulosa*, and *X. albilineans*). *X. albilineans*, *X. sacchari*, and *X. translucens* pv *undulosa* caused leaf scald of sugarcane, chlorotic leaf streak of sugarcane, and leaf streak of wheat, respectively.

**Figure 4:**
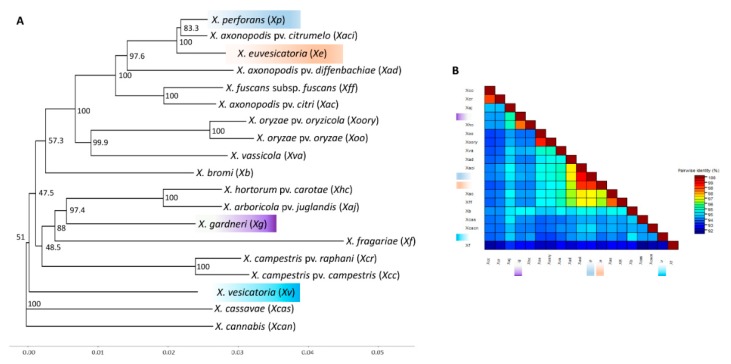
Phylogenetic positions of *Xanthomonas euvesicatoria* and *X. vesicatoria* and other *Xanthomonas* species using chromosomal replication initiator factor (*dnaA*), type I glyceraldehyde-3-phosphate dehydrogenase (*gapA*), DNA topoisomerase (ATP-hydrolyzing) subunit B (*gyrB*), hydroxymethylbilane synthase (*hmbs*), pyruvate dehydrogenase (*pdg*), molecular chaperone DnaK (*dnaK*), sugar ABC transporter permease (*lacF*), elongation factor G (*fusA*), and citrate synthase (*gltA*) genes. (**A**) Phylogenetic tree showing the positions of *Xanthomonas* species, (**B**) color-coded heat map generated from concatenated sequences of nine complete genes (mentioned above). Numbers on the nodes represent bootstrap values and are presented as a percentage of 1000 replicates. Line in the bottom represents the scale bar that shows the distance between the species.

To comprehensively determine the position *X. euvesicatoria* and *X. vesicatoria* in a phylogenetic tree, a concatenated tree was constructed using the complete sequences of nine genes (*dnaA*, *gyrB*, *gapA*, *pdg*, *hmbs*, *lacF*, *fusA*, *gltA*, *dnaK*) extracted from representative genomes of *Xanthomonas* species/subspecies/pathovars that cause diseases in different plants (Figure 4). The tree was constructed with a concatenated fragment 14,595 (14,601 including gaps) bp long. Among the four species of *Xanthomonas* causing bacterial spot, *X. euvesicatoria* and *X. perforans* were most closely related. They grouped together with *X. axonopodis* pv. *citrumelo*, a pathogen of citrus causing bacterial leaf spot and showed 99.1% and 98.6% similarity with *X. perforans* and *X. euvesicatoria*, respectively (Figure 4). *X. gardneri* was closest to *X. hortorum* pv. *carotae*, a pathogen of carrot causing bacterial leaf blight. *X. vesicatoria* did not show high similarity with *X. bromi*, as revealed in Figure 2, which was generated using five gene sequences, while Figure 4 was generated with nine gene sequences. Both trees (Figure 2 and Figure 4) reveal the same picture except that *X. vesicatoria* grouped with *X. bromi* in Figure 2, but *X. vesicatoria* grouped separately from all the species and away from *X. bromi* in Figure 4. Also, *X. euvesicatoria* grouped with *X. perforans* and *X. axonopodis* pv. *citrumelo* in Figure 4, while it was well separated from rest of the species in Figure 2.

**Figure 5:**
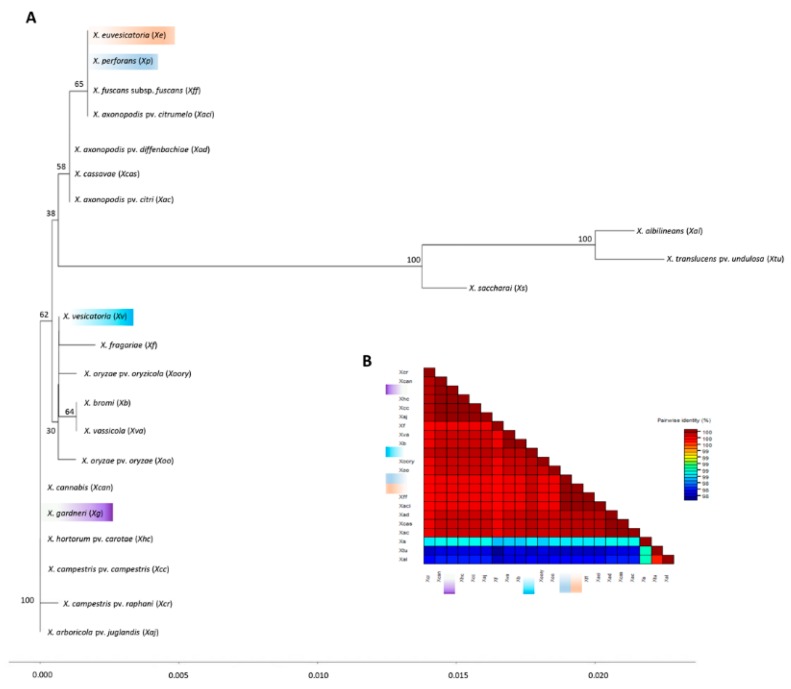
Phylogenetic analysis of species of plant pathogenic *Xanthomonas* (Table 3) using complete 16s rRNA gene sequence. (**A**) Phylogenetic tree showing the position of *Xanthomonas euvesicatoria* and *X. vesicatoria* within the other species of plant pathogenic *Xanthomonas.* (**B**) Color-coded matrix showing pairwise percentage identity of 16s rRNA sequences of *X. euvesicatoria* and *X. vesicatoria* with the other species of plant pathogenic *Xanthomonas*. Numbers on the nodes represent bootstrap values and are presented as the percentage of 1000 replicates. Line in the bottom represents the scale bar that shows the distance between the species.

Sequences for 16s rRNA exhibited less diversity among the plant pathogenic xanthomonads compared to the other genes used in the study. The phylogenetic tree obtained with 16s rRNA gene sequences was not congruent with the concatenated trees produced with the five genes (*dnaA*, *gyrB*, *hmbs*, *pdg*, and *gapA*) or nine genes (*dnaA*, *gyrB*, *gapA*, *pdg*, *hmbs*, *lacF*, *fusA*, *gltA*, *dnaK*). All *Xanthomonas* species of plant pathogenic xanthomonads included in this study resolved into three major clades. The 16s sequences for *X. euvesicatoria*, *X. perforans*, *X. fuscans* subsp. *fuscans*, and *X. axonopodis* pv. *citrumelo* were identical, so were the sequences for *X. axonopodis* pv. *diffenbachiae*, *X. cassavae*, and *X. axonopodis* pv. *citri.* These species grouped together to form a clade with *X. albilineans*, *X. sacchari*, and *X. translucens* pv. *undulosa* (Figure 5). *X. albilineans*, *X. saccharai*, and *X. translucens* pv. *undulosa* were very different from rest of the xanthomonads and grouped away with other species in this clade. *X. cannabis*, *X. gardneri*, *X. hortorum* pv. *carotae*, *X. campestris* pv. *campestris*, *X. raphani*, and *X. arboricola* pv. *juglandis* clustered together in another clade. The third clade was formed by *X. vesicatoria*, *X. fragariae*, *X. oryzae* pv. *oryzicola*, *X. bromi*, *X. vassicola*, *and X. oryzae* pv. *oryzae.*

### 3.4. Phylogenetic Analysis of Antibody Data

**Figure 6:**
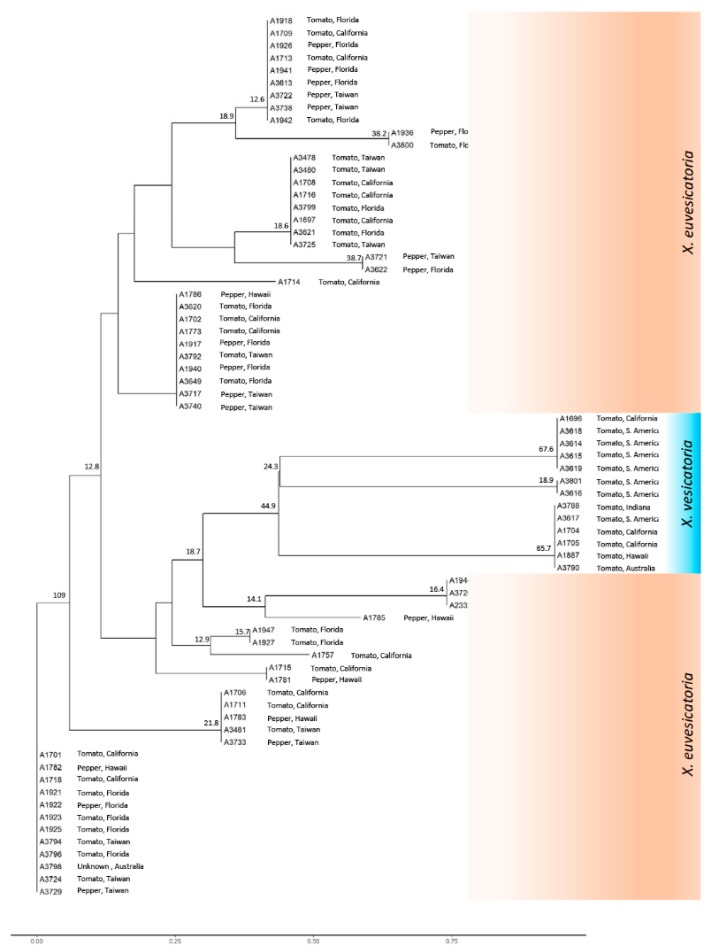
Phylogenetic tree for *Xanthomonas euvesicatoria* and *X. vesicatoria*, obtained using binary data for reactivity of these species with monoclonal antibodies Mab 1, Mab 3, Mab 5, Mab 7, Mab 8, and Mab 15. Bootstrap values shown on nodes are presented as the percentage of 1000 replicates. Line in the bottom represents the scale bar that shows the distance between the species.

In ELISA, monoclonal antibodies, Mab1, Mab3, Mab5, and Mab7 reacted specifically with *X. euvesicatoria*, whereas Mab8 or Mab15 reacted with the *X. vesicatoria* strains. None of the *X. vesicatoria* strains that reacted with Mab8 also reacted with Mab15. One *X. euvesicatoria* strain (A1757) reacted with Mab8, as well as with the *X. euvesicatoria*-specific Mabs (Mab1, 3, 5, and 7). For the phylogenetic analysis, a positive reaction with any antibody was scored 1 and a negative reaction was scored 0 to obtain binary data matrix. Upon analysis, a combination of Mabs 1, 3, 5, 7, 8, and 15 clearly separated *X. euvesicatoria* from *X. vesicatoria*. The *X. vesicatoria* strains grouped together in a single clade with three subgroups, while *X. euvesicatoria* formed 12 clades (Figure 6). Strains formed clusters independent of the geographic locations where they were collected. Nevertheless, most of the *X. vesicatoria* strains were from South America and California, and none were from Florida or Taiwan.

## 4. Discussion

MLSA with six genes (*hrcN*, *dnaA*, *gapA*, *gyrB*, *hmbs*, and *pdg*) and ELISA were used to characterize the diversity within and among *X. euvesicatoria* and *X. vesicatoria* collected from a wide range of geographic locations. Phylogenetic analysis with ELISA and all six individual genes resolved *X. euvesicatoria* and *X. vesicatoria* into different clusters (Figure 6 and Figures S1–S6). *X. vesicatoria* exhibited higher diversity and fewer geographical origins than *X. euvesicatoria*, while the latter strains showed less diversity but greater geographical representation. Genes *hmbs* and *pdg*, identified through comparative genomics, showed higher resolution within *X. euvesicatoria* than the genes traditionally used for population genetics studies of xanthomonads.

*X. euvesicatoria* had greater representation in our collection (58 out of 76 strains), but exhibited less diversity compared to *X. vesicatoria*. This may indicate that relatively uniform populations of *X. euvesicatoria* were spread through the contaminated seed. Group A1 strains with identical sequences were isolated from plants in California, Florida, and Taiwan, and group A2 strains were from California, Florida, Hawaii, Taiwan, and Australia. The presence of identical genotypes across Asia and America (group A1) and America, the Pacific, Asia, and Australia (group A2) clearly suggests that international seed trade played a significant role in spreading this BLS pathogen across the globe. Interestingly, *X. perforans*, first detected in Florida in 1991, was also not found in our collection—this indicates that this species emerged in Florida after 1990.

Surprisingly, the greatest genetic diversity was observed within the smaller number of *X. vesicatoria* (*n* = 14) strains evaluated in this study, rather than within *X. euvesicatoria* strains. Concatenated analysis of *X. vesicatoria* formed small subgroups within the larger *X. vesicatoria* clade; similar groups were formed when the phylogenetic tree was generated using individual genes. Higher diversity within *X. vesicatoria* was also reported by other researchers [8,17,27]. Geographic separation of groups within *X. vesicatoria* has also been reported by Timilsina et al. [17]. Most of the *X. vesicatoria* strains used in this study were from South America and California; they formed five small subgroups, whereas individual strains from Australia, Indiana, and Hawaii showed distinct genotypes and grouped separately from South American and Californian strains. *X. vesicatoria* strain A1887 from Hawaii was distinct from all the other *X. vesicatoria*. Two *X. vesicatoria* strains (A3618 and A3619) from South America were identical to A1696 from California; this also could be due to the movement of infected seeds from one location to the other.

Genes *hrcN*, *gyrB*, and *gapA* showed little resolving power within *X. euvesicatoria*, with the result that all *X. euvesicatoria* strains grouped into a single cluster (Figures S1, S3 and S4). Arif et al. [14] obtained no differences in populations of the ryegrass bacterial pathogen *Rathayibacter toxicus* when traditional housekeeping genes were used; however, the selection of the genes through comparative genomics resolved the populations into three distinct groups. In contrast to the previous report [17], *gapA* did not differentiate *X. euvesicatoria* populations; this could be due to different origins of the strains. We did not have access to the strains from India, Barbados, and Grenada, which formed a second group in the previous phylogenetic analysis [17]. Thus, it is likely that there are more than three populations of *X. euvesicatoria* worldwide; therefore, a comprehensive study with strains representing broader geographic locations is needed before the true diversity within *X. euvesicatoria* can be resolved. MLSA genes *dnaA*, *hmbs*, and *pdg* differentiated *X. euvesicatoria* strains into two groups; the two subgroups resolved using *hmbs* and *pdg* were identical and well supported with high bootstrap values (Figures S5 and S6). The *X. euvesicatoria* group A1 resolved using *hmbs* and *pdg* was host-specific, comprising strains from tomato only. Group A2 consisted mostly of pepper strains and some tomato strains. The host of strain A3798 (subgroup within group A2) was unknown to us when received.

The phylogenetic tree generated using the binary data from ELISA analysis showed a clear separation of the *X*. *vesicatoria* strains from the *X*. *euvesicatoria* strains (Figure 6). The ELISA analysis showed better resolution than MLSA and further divided the *X. euvesicatoria* strains into 13 subgroups (Figure 6). The greater diversity of individual populations can be attributed to the presence of multiple antigenic determinants on bacterial cell surfaces that trigger host responses in plant–bacterial interactions.

Phylogenetic analysis with all the genes except *pdg* placed *X. perforans* very close to *X. euvesicatoria*; *pdg* gene analysis showed that *X. perforans* shared 96.9–97.5% ANI with *X. vesicatoria*, while only 93.8%–94.1% ANI with *X. euvesicatoria* strains (Figure S6). Sequences of five genes (*dnaA*, *gyrB*, *gapA*, *hmbs*, and *pdg*) were extracted from a publicly available genome database of plant pathogenic xanthomonads (including *X. euvesicatoria* and *X. vesicatoria* reference strains) and an analysis was done to determine the position of BLS xanthomonads relative to these species and vice versa. Phylogenetic analysis placed *X. euvesicatoria*, *X. vesicatoria*, *X. gardneri*, and *X. perforans* in strongly supported clades (Figure 2). Separation of *X. euvesicatoria*, *X. vesicatoria*, and *X. gardneri* as different species is supported by phylogenetic analysis as well as ANI values. These species shared less than 94% ANI (Figure 3). There is an ongoing debate as to whether to separate *X. perforans* from *X. euvesicatoria*, with a group of researchers believing they should be placed together as a single species [18,28,29,30]. Interestingly, in our analysis, *X. perforans* was most closely related to *X*. *axonopodis* pv. *citrumelo* and these strains were placed close together in the phylogenetic analysis with strong bootstrap support (Figure 2 and Figure 4). *X. perforans* and *X. axonopodis* pv. *citrumelo* shared 99.2% common nucleotide sequences (Figure 3). *X. euvesicatoria* and *X. perforans* were 97.5% identical in these five gene sequences (Figure 3). More studies at genome level are needed to determine whether *X. perforans* is closer to *X. euvesicatoria* or *X. axonopodis* pv. *citrumelo*, and to determine whether these three species should be retained as a separate species or be combined. The *X. gardneri* strain grouped together with *X. hortorum* pv. *carotae* with 100% bootstrap support (97.7% ANI) and *X. vesicatoria* was placed together in a clade with *X. bromi* (94% ANI) (Figure 2 and Figure 3). Based on phylogenetic analysis of four housekeeping genes, Young et al. [30] proposed that *X. albilineans*, *X. sacchari*, *X. translucens* pv. *undolusa*, and *X. hyacinthi* might be a different genus. In our study, *X. albilineans*, *X. sacchari*, and *X. translucens* pv. *undolusa* were observed to be very different to rest of the *Xanthomonas* species and revealed less than 85%, 88%, and 89% ANI with rest of *Xanthomonas* species, respectively. The sequences of *dnaK*, *fusA*, *gltA*, and *lacF* genes were not matched when mapped with the *X. albilineans*, *X. sacchari*, and *X. translucens* pv. *undolusa* genomes available in the NCBI GenBank databases, which makes them different from other species. The similar positions were also revealed in 16s RNA gene analysis (Figure 5), however, 16s RNA analyses are not known for comprehensive discrimination at species level.

Recombination in the housekeeping genes *atpD* [27,31], *gapA*, and *gyrB* [17] has been reported earlier in *X. euvesicatoria* and *X. perforans.* The *atpD* gene was not used in this study and no recombination was observed in *gapA* and *gyrB* genes. Homologous recombination usually occurs between closely related species or pathovars within a species [32,33]. Rates of recombination and acquisition of genes by horizontal gene transfer are high among xanthomonads [34] and a minimum of 10% of the core genes are laterally transferred during evolution of gamma proteobacteria [35].

In conclusion, we have described the genetic diversity of *X. euvesicatoria* and *X. vesicatoria*, and the phylogenetic positions of *X. euvesicatoria* and *X. vesicatoria* relative to each other and other xanthomonads. Little genetic diversity among *X. euvesicatoria* strains is consistent with worldwide movement of clonal populations in seeds, whereas geographic isolation appears to be shaping the population structure of *X. vesicatoria.* MLST analyses also illustrated that *X. albilineans*, *X. sacchari*, and *X. translucens* pv. *undolusa* were very different to rest of the *Xanthomonas* species and showed less than 85%, 88%, and 89% ANI with rest of *Xanthomonas* species, respectively. Knowledge of the genetic diversity in bacterial populations is fundamental to disease management decisions at the field and policy level.

## Figures and Tables

**Table 1:** Details of the *Xanthomonas euvesicatoria*, *X. vesicatoria*, *X. gardneri*, and *X. perforans* strains used in the population genetics study.

A Number	Other ID	Origin	Host	Identity	Acquired Date
A1701	B94	California	Tomato	*Xanthomonas euvesicatoria*	1986
A1711	K625/B63	California	Tomato	*X. euvesicatoria*	1986
A3620	Xv 153	Florida	Tomato	*X. euvesicatoria*	1990
A1782	K337	Hawaii	Pepper	*X. euvesicatoria*	1986
A1781	K336	Hawaii	Pepper	*X. euvesicatoria*	1986
A1786	K339	Hawaii	Pepper	*X. euvesicatoria*	1986
A3480	XVT20	Taiwan	Tomato	*X. euvesicatoria*	1990
A3478	K348/XVT8	Taiwan	Tomato	*X. euvesicatoria*	1990
A1702	K618/B111	California	Tomato	*X. euvesicatoria*	1986
A1706	K622/B62	California	Tomato	*X. euvesicatoria*	1986
A1708	K623/B93	California	Tomato	*X. euvesicatoria*	1986
A1709	K624/B108	California	Tomato	*X. euvesicatoria*	1986
A1713	K626/B78	California	Tomato	*X. euvesicatoria*	1986
A1714	K627/B81	California	Tomato	*X. euvesicatoria*	1986
A1715	K628/B92	California	Tomato	*X. euvesicatoria*	1986
A1716	K629/B95	California	Tomato	*X. euvesicatoria*	1986
A1718	K630/B106	California	Tomato	*X. euvesicatoria*	1986
A1757	K641/XCV1	California	Tomato	*X. euvesicatoria*	1986
A1773	K645/XCV2	California	Tomato	*X. euvesicatoria*	1986
A1783	MCG	Hawaii	Pepper	*X. euvesicatoria*	1986
A1785	EWCII	Hawaii	Pepper	*X. euvesicatoria*	1986
A1917	62-8	Florida	Pepper	*X. euvesicatoria*	1986
A1918	65-2a	Florida	Tomato	*X. euvesicatoria*	1986
A3799	Xv158	Florida	Tomato	*X. euvesicatoria*	1991
A1921	69-13	Florida	Tomato	*X. euvesicatoria*	1986
A1922	71-21	Florida	Pepper	*X. euvesicatoria*	1986
A1923	71-39	Florida	Tomato	*X. euvesicatoria*	1986
A1925	75-4	Florida	Tomato	*X. euvesicatoria*	1986
A1926	77-3	Florida	Pepper	*X. euvesicatoria*	1986
A3794	Xv150	Taiwan	Tomato	*X. euvesicatoria*	1991
A3796	Xv155	Florida	Tomato	*X. euvesicatoria*	1991
A3792	Xv148	Taiwan	Tomato	*X. euvesicatoria*	1991
A3798	Xv157	Australia	NA	*X. euvesicatoria*	1991
A3800	Xv159	Florida	Tomato	*X. euvesicatoria*	1991
A1697	B79	California	Tomato	*X. euvesicatoria*	1986
A1936	82-12	Florida	Pepper	*X. euvesicatoria*	1986
A1940	82-16	Florida	Pepper	*X. euvesicatoria*	1986
A1941	82-17	Florida	Pepper	*X. euvesicatoria*	1986
A3621	Xv158	Florida	Tomato	*X. euvesicatoria*	1990
A3481	XVT14	Taiwan	Tomato	*X. euvesicatoria*	1990
A3613	Xv134	Florida	Pepper	*X. euvesicatoria*	1990
A3622	XV173	Florida	Pepper	*X. euvesicatoria*	1990
A3649	XV154	Florida	Tomato	*X. euvesicatoria*	1990
A3717	XVP28	Taiwan	Pepper	*X. euvesicatoria*	1991
A3721	XVP41	Taiwan	Sweet pepper	*X. euvesicatoria*	1991
A3722	XVP42	Taiwan	Sweet pepper	*X. euvesicatoria*	1991
A3724	XVT7	Taiwan	Tomato	*X. euvesicatoria*	1991
A3725	XVT8	Taiwan	Tomato	*X. euvesicatoria*	1991
A3726	XVT14	Taiwan	Tomato	*X. euvesicatoria*	1991
A3729	XVP1	Taiwan	Sweet pepper	*X. euvesicatoria*	1991
A3733	XVP5	Taiwan	Sweet pepper	*X. euvesicatoria*	1991
A3738	XVP10	Taiwan	Sweet pepper	*X. euvesicatoria*	1991
A3740	XVP12	Taiwan	Sweet pepper	*X. euvesicatoria*	1991
A1927	80-1	Florida	Tomato	*X. euvesicatoria*	1986
A1942	83-4	Florida	Tomato	*X. euvesicatoria*	1986
A1944	83-13	Florida	Tomato	*X. euvesicatoria*	1986
A1947	E3	Florida	Tomato	*X. euvesicatoria*	1986
A2332	X298	Florida	Tomato	*X. euvesicatoria*	1990
A3617	XV145	S. America	Tomato	*X. vesicatoria*	1990
A3616	XV144	S. America	Tomato	*X. vesicatoria*	1990
A3618	XV146	S. America	Tomato	*X. vesicatoria*	1990
A3788	CC12, Xv138	Indiana	Tomato	*X. vesicatoria*	1991
A1696	K613/B71	California	Tomato	*X. vesicatoria*	1986
A1703	K619/B118	California	Tomato	*X. vesicatoria*	1986
A1704	K620/B122	California	Tomato	*X. vesicatoria*	1986
A1705	K621/XV-1	California	Tomato	*X. vesicatoria*	1986
A1887	K663/A135-1	Hawaii	Tomato	*X. vesicatoria*	1986
A3801	Xv142a	S. America	Tomato	*X. vesicatoria*	1991
A3790	Xv140	Australia	Tomato	*X. vesicatoria*	1991
A3614	XV142b	S. America	Tomato	*X. vesicatoria*	1990
A3615	XV143	S. America	Tomato	*X. vesicatoria*	1990
A3619	XV147	S. America	Tomato	*X. vesicatoria*	1990
	Xg-51	Canada	Tomato	*X. gardneri*	*
	Xg-444	Costa-Rica	Tomato	*X. gardneri*	*
	Gev 4E5	Florida	Tomato	*X. perforans*	*
	91-118	Florida	Tomato	*X. perforans*	*

MCG: Manoa community garden; NA: host information not available; K numbers refer to strains characterized using the *dnaA* RIF marker by Schneider et al. [19]; acquired date (when we received the strain) is not an isolation date; * we received only DNA, not the strains; 91-118 was isolated in 1991 [17]. For a few strains we have date of isolation: 62-8 in 1962; 65-2a in 1965; 69-13 in 1969; 71-21 and 71-39 in 1971; 75-4 in 1975; 77-3 in 1977; 82-12, 82-16, and 82-17 in 1982; 80-1 in 1980; 83-4 and 83-13 in 1983; for all Hawaiian strains acquired and isolation dates are the same.

**Table 2:** Details of the primers used for PCR amplification of EscN/YscN/HrcN family type III secretion system ATPase (*hrcN*), chromosomal replication initiator factor (*dnaA*), DNA topoisomerase (ATP-hydrolyzing) subunit B (*gyrB*), type I glyceraldehyde-3-phosphate dehydrogenase (*gapA*), hydroxymethylbilane synthase (*hmbs*), and pyruvate dehydrogenase (*pdg*) gene.

Target Gene	Primers Name	Primer Sequences (5′-3′)	Product Size
*hrcN*	X-hrcN-F	TCGGCACCATGCTCAAGGT	846
	X-hrcN-F	GTGTAGAACGCGGTGATCGA	
*dnaA*	dnaA-F	CAGCACGGTGGTGTGGTC	928
	dnaA-R	CCTGGATTCGCATTACACC	
*gyrB*	GyrB-F2	GAGGTGATCCTCACCGTGCT	841
	GyrB-R2	TGATGGCCTTGGCTTCGTTC	
*gapA*	gapA-F1	TGGCCATCAATGACCTGCTC	865
	gapA-R1	TAGCCCCACTCGTTGTCGTA	
*pdg*	pdg-F	CCACCCACCAGACCAAGAA	990
	pdg-R	CAGGTACATGCCCTTGATGA	
*hmbs*	Hmbs-F	GTATCGCCACCCGCAAAA	873
	Hmbs-R	CCTTGTCGAACAGCCCTTG	
	Hmbs-F2	TTGCATCGCCACCCGCAAGA	837
	Hmbs-R2	TCCTTGTCGAACAGGCCTTG	
	Hmbs-F10	AGGGCCTGTTTTTGAAGGAA	595
	Hmbs-R10	AACCCCTCGCCTTCCCAGGT	

**Table 3:** Details of the xanthomonads genomes used to retrieve the gene sequences for phylogenetic analysis.

Species	Accession Numbers	Host	Geographic Location
*X. perforans*	NZ_CP019725	Tomato	Mauritius
*X. gardneri*	NZ_CP018731	Tomato	New Zealand
*X. vesicatoria*	NZ_CP018470	Tomato	New Zealand
*X. euvesicatoria*	NZ_CP018467	Pepper	USA
*X. fragariae*	NZ_CP016830	Strawberry	California, USA
*X. axonopodis* pv. *diffenbachiae*	NZ_CP014347	Anthurium	Brazil
*X. sacchari*	NZ_CP010409	Rice	China
*X. translucens* pv. *undulosa*	NZ_CP008714	Wheat	Kansas, USA
*X. hortum* pv. *carotae*	NZ_CM002307	Carrot	Oregon. USA
*X. fuscans* subsp. *fuscans*	NC_022541	Bean	France
*X. campestris* pv. *raphani*	NC_017271	Cabbage	East Asia
*X. oryzae* pv. *oryzicola*	NC_017267	Rice	Philippines
*X. axonopodis* pv. *citrumelo*	NC_016010	Citrus	Florida, USA
*X. albilineans*	NC_013722	Sugarcane	France
*X. oryzae* pv. *oryzae*	NC_007705	Rice	Japan
*X. axonopodis* pv. *citri*	NC_003919	Mexican lime	Florida, USA
*X. campestris pv. campestris*	NC_003902	Cabbage	UK
*X. arboricola* pv. *juglandis*	CP012251	Walnut	USA
*X. bromi*	GCF_900092025.1	Brome grass	France
*X. cannabis*	GCF_000802405.1	*Cannabis sativa*	Japan
*X. vassicola*	GCF_000772705.2	Sorghum	New Zealand
*X. cassavae*	GCF_000454545.1	Cassava	Malawi

**Table 4:** Details of the monoclonal antibodies (Mabs) used to characterize *Xanthomonas euvesicatoria* and *X. vesicatoria.*

Mab Name	Clone Number	Subclass	Immunogen (s)
Xv1	106-41-1-1	IgG2b	A1074
Xv3	131-39-14-2	IgG2b	82-17
Xv5 *	131-10-9-1	IgG3	82-17
Xv7 *	130-10-2-1	NA	65-2a
Xv8	4H5-3B1	IgG1	BA29-1, BV20-3A, X525-85
Xcv15	209-C15-4-4	IgM	B61, B80

Mabs Xv 1, 3, 8, and 15 were used in a previous survey of *X*. *campestris* pv. *vesicatoria* [6]; * Mabs Xv5 and Xv7 were produced from hybridoma cell lines previously generated at the University of Hawaii and data from these Mabs were not included in the analysis by Bouzar, et al., [6].

## Supplementary Materials

The following are available online at https://www.mdpi.com/2076-2607/7/10/462/s1.
