# Supplementary material for: Phylogenetic Analyses of Xanthomonads Causing Bacterial Leaf Spot of Tomato and Pepper: Xanthomonas euvesicatoria Revealed Homologous Populations Despite Distant Geographical Distribution
Source: Microorganisms. 2019 Oct 16;7(10):462. doi: 10.3390/microorganisms7100462 (PMC6843189; doi:10.3390/microorganisms7100462)
Supplement: Supplementary file 1 [file microorganisms-07-00462-s001.pdf]

Supplementary Materials

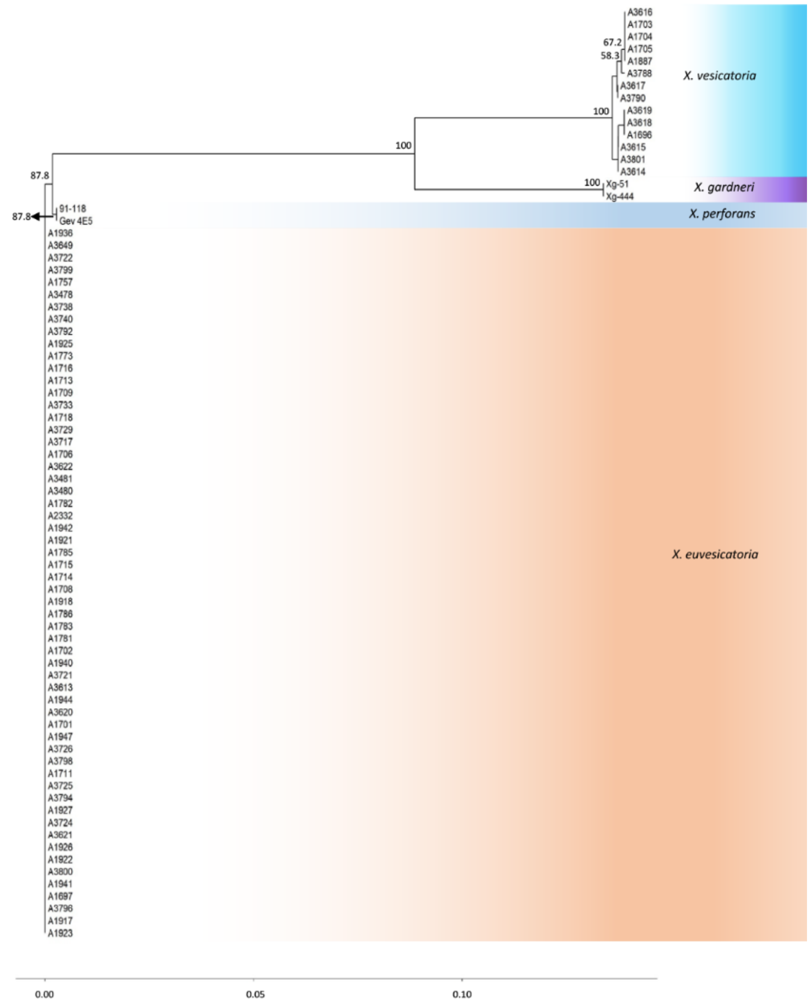

Figure S1. Phylogenetic analysis of *Xanthomonas euvesicatoria*, *X. vesicatoria*, *X. gardenri,* and *X. perforans* using *hrcN* gene. Strain information is provided in Table 1. Numbers on the nodes represent bootstrap values and are presented as percentage of 1000 replicates. Line in the bottom represents the scale bar that shows the distance between the species.

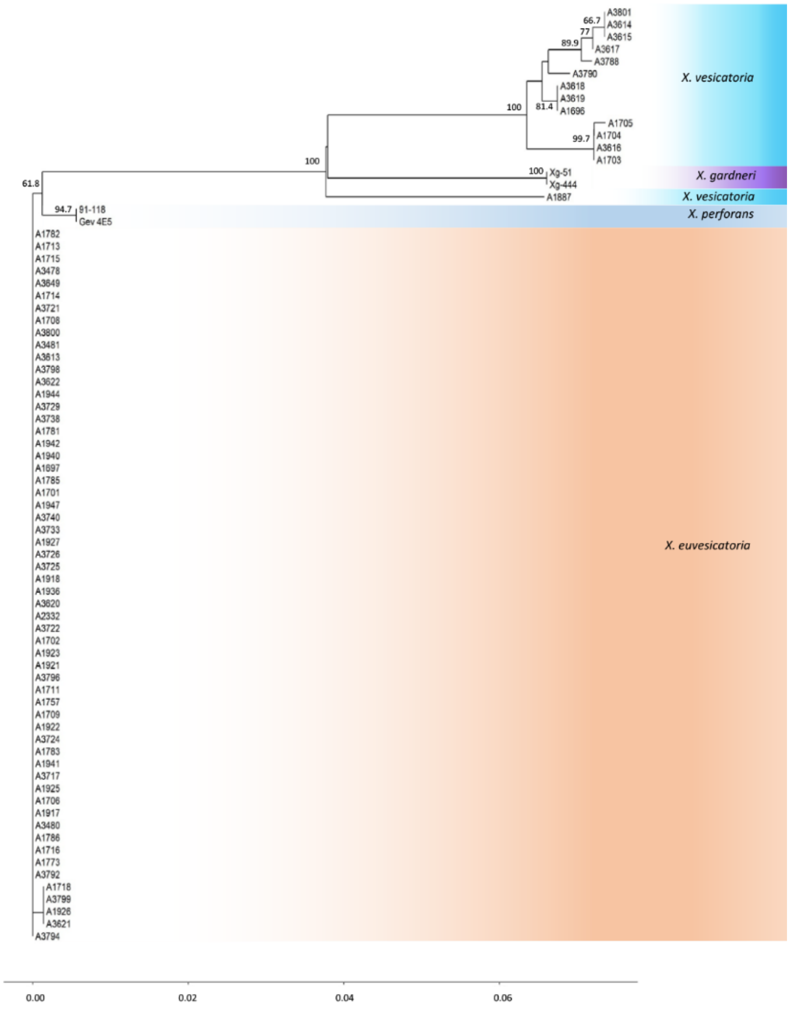

Figure S2. Phylogenetic analysis of *Xanthomonas euvesicatoria*, *Xanthomonas vesicatoria*, *X. gardeneri,* and *X. perforans* using chromosomal replication initiator factor (*dnaA*) gene. Strain information is provided in Table 1. Numbers on the nodes represent bootstrap values and are presented as percentage of 1000 replicates. Line in the bottom represents the scale bar that shows the distance between the species.

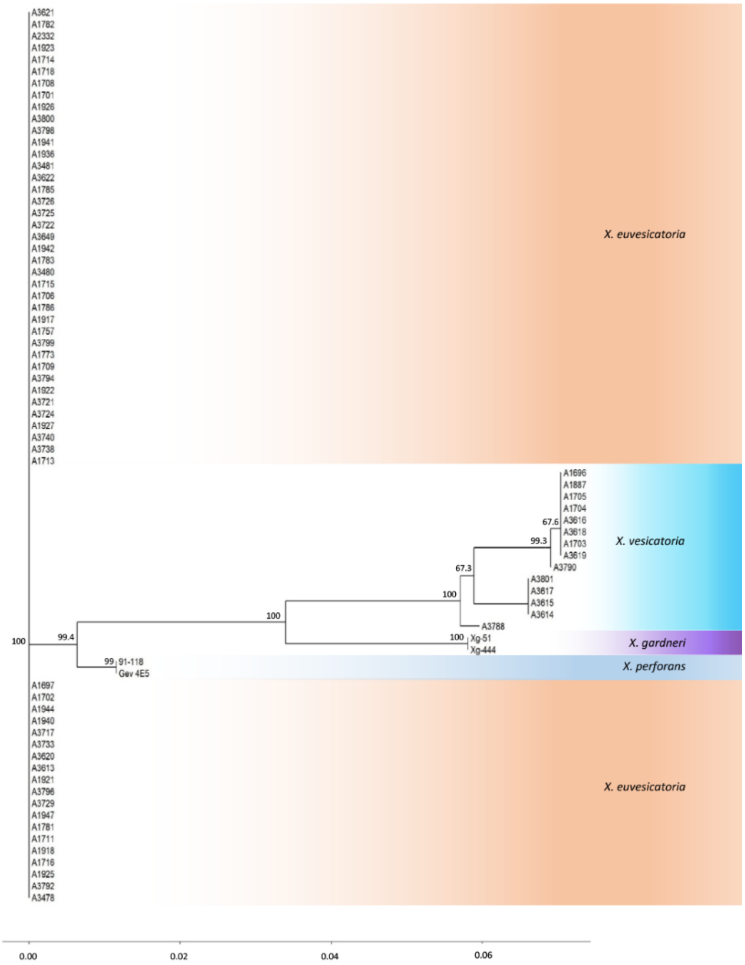

Figure S3. Phylogenetic analysis of *Xanthomonas euvesicatoria*, *Xanthomonas vesicatoria*, *X. gardenri,* and *X. perforans* using DNA topoisomerase (ATP-hydrolyzing) subunit B (*gyrB*) gene. Strain information is provided in Table 1. Numbers on the nodes represent bootstrap values and are presented as percentage of 1000 replicates. Line in the bottom represents the scale bar that shows the distance between the species.

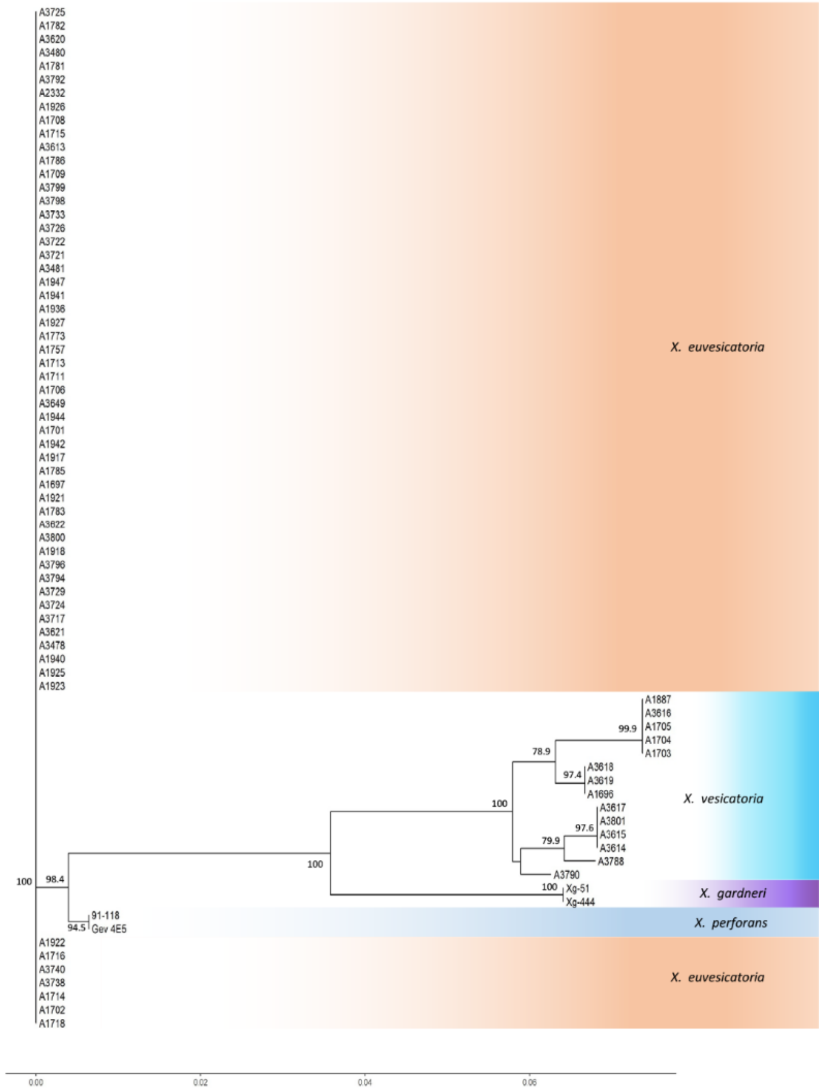

Figure S4. Phylogenetic analysis of *Xanthomonas euvesicatoria*, *Xanthomonas vesicatoria*, *X. gardenri*, and *X. perforans* using type I glyceraldehyde-3-phosphate dehydrogenase (*gapA*) gene. Strain information is provided in Table 1. Numbers on the nodes represent bootstrap values and are presented as percentage of 1000 replicates. Line in the bottom represents the scale bar that shows the distance between the species.

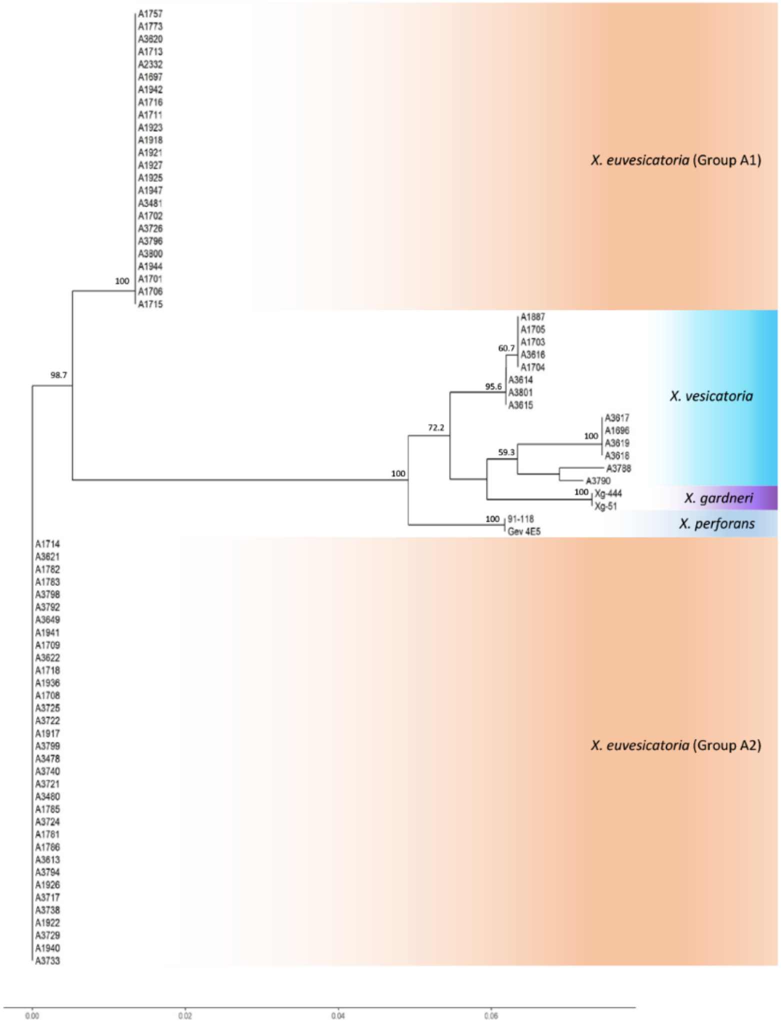

Figure S5. Phylogenetic analysis of *Xanthomonas euvesicatoria* and *Xanthomonas vesicatoria* using pyruvate dehydrogenase (*pdg*) gene. Strain information is provided in Table 1. Numbers on the nodes represent bootstrap values and are presented as percentage of 1000 replicates. Line in the bottom represents the scale bar that shows the distance between the species.

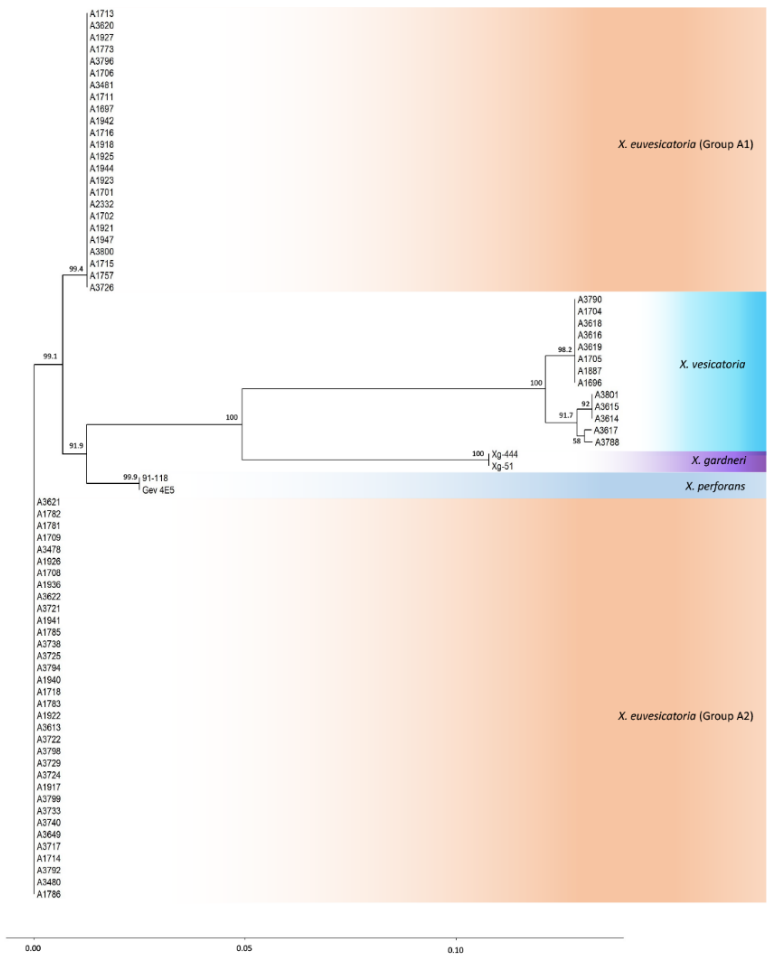

Figure S6. Phylogenetic analysis of *Xanthomonas euvesicatoria*, *Xanthomonas vesicatoria*, *X. gardneri*, and *X. perforans* using (*hmbs*) gene. Strain information is provided in Table 1. Numbers on the nodes represent bootstrap values and are presented as percentage of 1000 replicates. Line in the bottom represents the scale bar that shows the distance between the species.

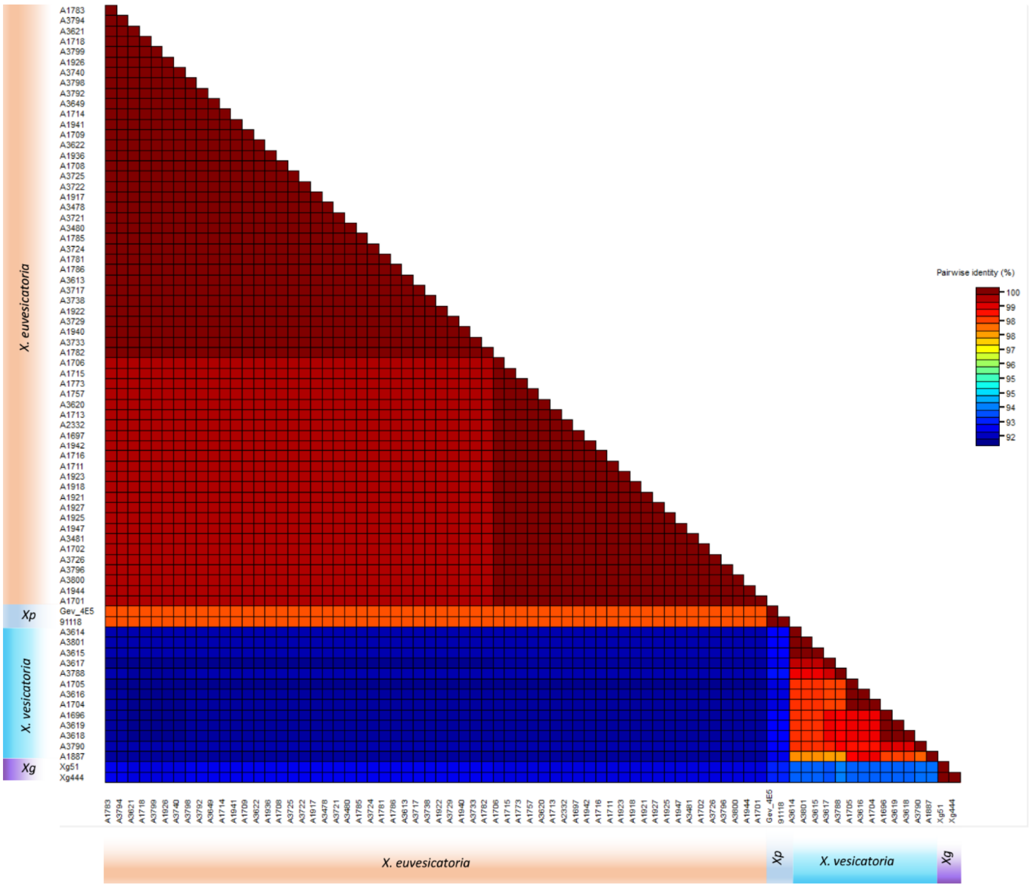

Figure S7. Color-coded matrix showing pairwise percentage identity of *X. euvesicatoria, X. vesicatoria, X. perforans,* and *X. gardneri* isolates used in the study for using EscN/YscN/HrcN family type III secretion system ATPase (*hrcN*), chromosomal replication initiator factor (*dnaA*), DNA topoisomerase (ATP-hydrolyzing) subunit B (*gyrB*), type I glyceraldehyde-3-phosphate dehydrogenase (*gapA*), hydroxymethylbilane synthase (*hmbs*), and pyruvate dehydrogenase (*pdg*) genes. Table S1**:** List of GenBank accession numbers for the strains sequenced for genes EscN/YscN/HrcN family type III secretion system ATPase (*hrcN*), chromosomal replication initiator factor (*dnaA*), DNA topoisomerase (ATP-hydrolyzing) subunit B (*gyrB*), type I glyceraldehyde-3-phosphate dehydrogenase (*gapA*), pyruvate dehydrogenase (*pdg*), and hydroxymethylbilane synthase (*hmbs*).

Table S1: List of GenBank accession numbers for the strains sequenced for genes, EscN/YscN/HrcN family type III secretion system ATPase (*hrcN*), chromosomal replication initiator factor (*dnaA*), DNA topoisomerase (ATP-hydrolyzing) subunit B (*gyrB*), type I glyceraldehyde-3-phosphate dehydrogenase (*gapA*), pyruvate dehydrogenase (*pdg*) and hydroxymethylbilane synthase (*hmbs*).

Strain ID	Strain	*gyrB*	*pdg*	*hmbs*	*dnaA*	*gapA*	*hrcN*
A1701	*Xanthomonas euvesicatoria*	MH481384	MH484500	MH492100	MH492152	MH492218	MG847408*
A1711	*X. euvesicatoria*	MH481429	MH484487	MH492092	MH492168	MH492214	MG847400*
A3620	*X. euvesicatoria*	MH481422	MH484481	MH492085	MH492161	MH492189	MH510054
A1782	*X. euvesicatoria*	MH481375	MH484443	MH492051	MH492112	MH492187	MH510047
A1781	*X. euvesicatoria*	MH481428	MH484468	MH492053	MH492147	MH492191	MG847392*
A1786	*X. euvesicatoria*	MH481402	MH484469	MH492084	MH492180	MH492198	MG847389*
A3480	*X. euvesicatoria*	MH481399	MH484465	MH492083	MH492179	MH492190	MG847376*
A3478	*X. euvesicatoria*	MH481434	MH484462	MH492055	MH492134	MH492234	MG847378*
A1702	*X. euvesicatoria*	MH481417	MH484495	MH492102	MH492164	MH492243	MG847407*
A1706	*X. euvesicatoria*	MH481401	MH484445	MH492090	MH492177	MH492215	MG847403*
A1708	*X. euvesicatoria*	MH481383	MH484457	MH492057	MH492138	MH492195	MG847402*
A1709	*X. euvesicatoria*	MH481407	MH484453	MH492054	MH492170	MH492199	MG847401*
A1713	*X. euvesicatoria*	MH481415	MH484482	MH492086	MH492114	MH492213	MG847399*
A1714	*X. euvesicatoria*	MH481381	MH484451	MH492081	MH492136	MH492242	MG847398*
A1715	*X. euvesicatoria*	MH481400	MH484446	MH492106	MH492133	MH492196	MG847397*
A1716	*X. euvesicatoria*	MH481431	MH484486	MH492095	MH492181	MH492239	MG847396*
A1718	*X. euvesicatoria*	MH481382	MH484455	MH492067	MH492110	MH492244	MG847395*
A1757	*X. euvesicatoria*	MH481404	MH484480	MH492107	MH492169	MH492212	MG847394*
A1773	*X. euvesicatoria*	MH481406	MH484479	MH492088	MH492182	MH492211	MG847393*
A1783	*X. euvesicatoria*	MH481398	MH484447	MH492068	MH492173	MH492224	MG847391*
A1785	*X. euvesicatoria*	MH481392	MH484466	MH492062	MH492151	MH492221	MG847390*
A1917	*X. euvesicatoria*	MH481403	MH484460	MH492075	MH492178	MH492220	MH510069
A1918	*X. euvesicatoria*	MH481430	MH484489	MH492096	MH492159	MH492227	MG847386*
A3799	*X. euvesicatoria*	MH481405	MH484461	MH492076	MH492109	MH492200	MG847367*
A1921	*X. euvesicatoria*	MH481424	MH484490	MH492103	MH492166	MH492223	MG847385*
A1922	*X. euvesicatoria*	MH481409	MH484475	MH492069	MH492171	MH492238	MG847384*
A1923	*X. euvesicatoria*	MH481378	MH484488	MH492099	MH492165	MH492237	MG847383*
A1925	*X. euvesicatoria*	MH481432	MH484492	MH492097	MH492176	MH492236	MG847381*
A1926	*X. euvesicatoria*	MH481385	MH484472	MH492056	MH492111	MH492194	MH510063
A3794	*X. euvesicatoria*	MH481408	MH484471	MH492065	MH492184	MH492229	MH510059
A3796	*X. euvesicatoria*	MH481425	MH484497	MH492089	MH492167	MH492228	MH510068
A3792	*X. euvesicatoria*	MH481433	MH484449	MH492082	MH492183	MH492192	MH510041
A3798	*X. euvesicatoria*	MH481387	MH484448	MH492072	MH492142	MH492201	MH510057
A3800	*X. euvesicatoria*	MH481386	MH484498	MH492105	MH492139	MH492226	MH510064
A1697	*X. euvesicatoria*	MH481416	MH484484	MH492093	MH492150	MH492222	MH510066
A1936	*X. euvesicatoria*	MH481389	MH484456	MH492058	MH492160	MH492209	MH510036
A1940	*X. euvesicatoria*	MH481419	MH484477	MH492066	MH492149	MH492235	MH510050
A1941	*X. euvesicatoria*	MH481388	MH484452	MH492061	MH492174	MH492208	MH510065
A3621	*X. euvesicatoria*	MH481376	MH484444	MH492052	MH492113	MH492233	MH510062
A3481	*X. euvesicatoria*	MH481390	MH484494	MH492091	MH492140	MH492206	MH510046

A3613	*X. euvesicatoria*	MH481423	MH484470	MH492070	MH492141	MH492197	MH510052
A3622	*X. euvesicatoria*	MH481391	MH484454	MH492059	MH492143	MH492225	MH510045
A3649	*X. euvesicatoria*	MH481396	MH484450	MH492079	MH492135	MH492216	MH510037
A3717	*X. euvesicatoria*	MH481420	MH484473	MH492080	MH492175	MH492232	MH510044
A3721	*X. euvesicatoria*	MH481410	MH484464	MH492060	MH492137	MH492205	MH510051
A3722	*X. euvesicatoria*	MH481395	MH484459	MH492071	MH492163	MH492204	MH510038
A3724	*X. euvesicatoria*	MH481411	MH484467	MH492074	MH492172	MH492231	MH510061
A3725	*X. euvesicatoria*	MH481394	MH484458	MH492064	MH492158	MH492188	MH510058
A3726	*X. euvesicatoria*	MH481392	MH484496	MH492108	MH492157	MH492203	MH510056
A3729	*X. euvesicatoria*	MH481426	MH484476	MH492073	MH492145	MH492230	MH510043
A3733	*X. euvesicatoria*	MH481421	MH484478	MH492077	MH492155	MH492202	MH510042
A3738	*X. euvesicatoria*	MH481414	MH484474	MH492063	MH492146	MH492241	MH510039
A3740	*X. euvesicatoria*	MH481413	MH484463	MH492078	MH492154	MH492240	MH510040
A1927	*X. euvesicatoria*	MH481412	MH484491	MH492087	MH492156	MH492210	MH510060
A1942	*X. euvesicatoria*	MH481397	MH484485	MH492094	MH492148	MH492219	MH510049
A1944	*X. euvesicatoria*	MH481418	MH484499	MH492098	MH492144	MH492217	MH510053
A1947	*X. euvesicatoria*	MH481427	MH484493	MH492104	MH492153	MH492207	MH510055
A2332	*X. euvesicatoria*	MH481377	MH484483	MH492101	MH492162	MH492193	MH510048
A3617	*X. vesicatoria*	MH481447	MH484512	MH492046	MH492125	MH492258	MG847372*
A3616	*X. vesicatoria*	MH481441	MH484509	MH492040	MH492118	MH492247	MG847373*
A3618	*X. vesicatoria*	MH481440	MH484514	MH492041	MH492122	MH492253	MG847371*
A3788	*X. vesicatoria*	MH481446	MH484516	MH492045	MH492124	MH492256	MG847369*
A1696	*X. vesicatoria*	MH481443	MH484511	MH492034	MH492123	MH492254	MG847409*
A1703	*X. vesicatoria*	MH481439	MH484508	NA	MH492119	MH492251	MG847406*
A1704	*X. vesicatoria*	MH481444	MH484510	MH492038	MH492116	MH492250	MG847405*
A1705	*X. vesicatoria*	MH481445	MH484506	MH492036	MH492117	MH492249	MG847404*
A1887	*X. vesicatoria*	MH481442	MH484507	MH492035	MH492115	MH492248	MG847387*
A3801	*X. vesicatoria*	MH481448	MH484504	MH492043	MH492127	MH492257	MH510067
A3790	*X. vesicatoria*	MH481438	MH484515	MH492039	MH492120	MH492255	MG847368*
A3614	*X. vesicatoria*	MH481450	MH484503	MH492044	MH492126	MH492260	MG847375*
A3615	*X. vesicatoria*	MH481449	MH484505	MH492042	MH492128	MH492259	MG847374*
A3619	*X. vesicatoria*	MH481437	MH484513	MH492037	MH492121	MH492252	MG847370*
Xg-51	*X. gardneri*	MH481436	MH484517	MH492047	MH492130	MH492246	MG847357*
Xg-444	*X. gardneri*	MH481435	MH484518	MH492048	MH492129	MH492245	MG847356*
Gev4E5	*X. perforans*	MH481379	MH484501	MH492049	MH492131	MH492185	MG847358*
91-118	*X. perforans*	MH481380	MH484502	MH492050	MH492132	MH492186	MG847412*

NA: Isolate 1703 failed to provide specific product with the primers used, thus, no sequence is available. *refers to the sequences reported earlier in Larrea-Sarmiento et al (2018).
